# 多孔框架材料固定化酶生物传感器用于农药残留分析的研究进展

**DOI:** 10.3724/SP.J.1123.2026.03022

**Published:** 2026-11-08

**Authors:** Zhengyu LAN, Xiaoxue KOU, Jing LI, Tianyi WEN, Rui GAO, Siming HUANG, Guosheng CHEN, Gangfeng OUYANG

**Affiliations:** 1. 成都大学食品与生物工程学院，四川 成都 610106; 1. College of Food and Biological Engineering，Chengdu University，Chengdu 610106，China; 2. 中山大学化学学院，广东 广州 510275; 2. School of Chemistry，Sun Yat-sen University，Guangzhou 510275，China; 3. 成都大学高等研究院，四川 成都 610106; 3. Institute for Advanced Study，Chengdu University，Chengdu 610106，China; 4. 广州医科大学药学院，广东 广州 511436; 4. School of Pharmaceutical Sciences and the Fifth Aﬃliated Hospital，Guangzhou Medical University，Guangzhou 511436，China

**Keywords:** 多孔框架材料, 酶固定化, 农药, 监测, porous framework materials, enzyme immobilization, pesticides, monitoring

## Abstract

农药残留对生态系统安全和人类健康构成持续威胁，亟需发展兼具高灵敏度与便携性的快速检测技术。酶生物传感器虽具备优异的分子识别能力，但其实际应用常受限于天然酶固有的稳定性问题。多孔框架材料，包括金属有机框架、共价有机框架与氢键有机框架材料，凭借其超高比表面积、可调的孔道结构及良好的化学稳定性为酶的固定化提供了理想载体，可显著提升酶的催化活性、操作稳定性及底物选择性。本文系统综述了基于多孔框架材料构建的固定化酶生物传感器在农药监测中的研究进展。首先，归纳了表面吸附、后渗透与原位固定3类酶固定化方法，探讨了其在负载效率、稳定性及选择性方面的特征与适用性；进而，详细阐述了基于比色、荧光、电化学及化学发光等信号转导机制的传感模式，分析了其响应原理与检测性能。最后，针对复杂基质耐受性、多残留同步识别、绿色合成以及智能传感系统集成等现场应用中面临的关键挑战，提出了未来发展方向，旨在为开发高可靠性、适应复杂场景的农药快速检测技术提供理论依据与设计参考。

## Abstract (en)

Pesticide residues pose a persistent threat to ecosystem safety and human health，creating an urgent need for rapid detection techniques that combine high sensitivity with portability. Enzyme-based biosensors，with their excellent molecular recognition capabilities，have emerged as promising tools； however，their practical application is often hampered by the inherent instability of natural enzymes，including susceptibility to denaturation，limited operational pH and temperature ranges，and poor reusability. In this context，porous framework materials—specifically metal-organic frameworks，covalent organic frameworks，and hydrogen-bonded organic frameworks—have attracted considerable attention as ideal supports for enzyme immobilization. These materials offer ultrahigh specific surface areas，tunable pore architectures，and robust chemical stability，which together can significantly enhance enzyme catalytic activity，operational stability，and substrate selectivity via confinement effects，protective shielding，and microenvironment modulation. This review systematically summarizes recent progress in the development of immobilized-enzyme biosensors based on porous framework materials for pesticide monitoring. We first categorize three main enzyme immobilization strategies： surface adsorption，post-synthetic infiltration，and in situ encapsulation. Surface adsorption attaches enzymes via weak interactions，offering simplicity but potential leaching； post-synthetic infiltration exploits pore size matching to achieve high loading； and in situ encapsulation entraps enzymes during framework growth，conferring superior protection. For each strategy，we discuss characteristic features regarding loading efficiency，enzyme stability，and substrate selectivity，as well as applicability to different enzyme-framework combinations. We then provide a detailed account of sensing modalities based on colorimetric，fluorescent，electrochemical，and chemiluminescent signal transduction mechanisms. Colorimetric sensors utilize enzyme inhibition to produce visible signals，fluorescent sensors rely on signal changes from confined fluorophores，electrochemical platforms transduce enzymatic reactions into electrical outputs，and chemiluminescent systems offer low-background light emission. The underlying response principles，signal amplification strategies，and analytical performance metrics—such as detection limits，linear ranges，and selectivity—are critically examined. Furthermore，we address key challenges hindering on-site deployment： tolerance to complex sample matrices （e.g.，fruit juices，soil extracts） that cause nonspecific interferences； simultaneous recognition of multiple pesticide residues requiring multi-enzyme cascades or array-based differentiation； green and scalable synthesis of framework-enzyme composites under mild conditions； and integration into intelligent sensing systems （e.g.，smartphone-based readers，microfluidics，and wireless data transmission）. Finally，we propose future research directions aimed at overcoming these obstacles. By bridging materials chemistry and analytical science，this review is intended to provide a theoretical basis and design references for the development of highly reliable，field-deployable rapid detection technologies for pesticide residues，ultimately contributing to food safety and environmental protection.

农药是保障农业增产和农民增收的关键投入品，在农业生产中占据重要地位。过去30年来，随着全球人口增长和对农产品需求的持续增加，农药使用量逐年攀升。据联合国粮农组织统计，截至2022年，全球农药使用量已达370万吨^［1］^。然而，长期的过量使用与不合理施用导致农药残留问题日益严峻，对生态系统和人类健康构成严重威胁。进入环境的农药通过大气循环扩散至土壤和水体，易被作物根系吸收富集，并最终通过食物链在人体内蓄积。研究表明，农药暴露与神经毒性、免疫毒性、肝肾损伤及生殖毒性密切相关，并可诱发癌症、心血管疾病及胎儿畸形等病症^［2］^。因此，开发高灵敏、高特异性、便携式且低成本的快速检测技术，已成为全球食品安全筛查和环境污染监测领域的重大战略需求。

以气相色谱法^［3］^、高效液相色谱法^［4］^及其与质谱联用技术（如气相色谱-质谱联用技术^［5］^、液相色谱-质谱联用技术^［6］^）为代表的经典分析技术，凭借其适用范围广、分离效能高、灵敏度强及重复性好等优势，目前仍是复杂基质中痕量农药残留确证分析的“黄金标准”。然而，这类技术在实际检测流程中严重依赖于专业实验室环境，不仅仪器设备昂贵、笨重，且样品前处理过程烦琐耗时。上述瓶颈直接影响了粮食与农产品安全监测的时效性及现场快速响应能力，难以满足市场对农药残留快速筛查技术的迫切需求。

酶生物传感器因其出色的分子识别特异性、快速的响应能力以及易于微型化集成等优势，已在农药残留的快速检测领域展现出巨大的应用潜力^［7-9］^。在该类传感器中，酶作为核心生物识别元件，通过与目标分析物发生高特异性生化反应，产生物理或化学变化；这些变化随后被信号转换器捕获并转导为可定量输出的电学或光学信号。该技术深度融合了酶的高催化效率与“锁-钥”式的特异性识别机制，为实现复杂基质中痕量农药的高选择性分析提供了有效手段。然而，天然酶的固有结构不稳定性制约其广泛应用^［10］^。在实际检测场景中，样品基质往往成分复杂，例如富含有机酸和色素的果汁或pH波动较大的环境水样。酶分子在此类理化胁迫下极易发生三维构象的解折叠与不可逆变性^［7，11］^。这种构象的脆弱性导致了传感器信号的漂移、有效使用寿命的缩短以及批间重现性的下降，严重影响了酶生物传感器的检测准确性、长期可靠性及其实际应用的经济效益^［8］^。

近年来，如何有效提升酶的稳定性已成为生物催化领域亟待解决的关键科学问题。酶固定化技术通过将酶锚定于固体载体，不仅能够在复杂环境中维持其活性构象，还可显著增强酶对温度、pH及有机溶剂等环境胁迫因子的耐受性，同时实现酶的回收与重复利用，从而降低使用成本^［12，13］^。在该技术中，载体材料的性质对固定化效果具有决定性影响。多孔框架材料的出现为酶固定化提供了新的解决方案^［14-16］^。其中，金属有机框架（MOFs）是由金属离子或金属簇与有机配体通过配位键自组装形成的晶态多孔材料^［17］^；而共价有机框架（COFs）则由有机分子通过共价键连接而成；氢键有机框架（HOFs）则通过分子间氢键、*π-π*堆积等弱相互作用构筑而成。这3类材料凭借其超高的比表面积、可调的孔道结构、丰富的功能化位点以及良好的热稳定性与化学稳定性，已成为酶固定化的重要载体^［18，19］^。从分析传感的角度来看，多孔框架材料在构建酶生物传感器方面具备多重优势。首先，其刚性多孔骨架可作为高效保护性基质，通过空间限域效应稳定酶分子天然构象、抑制变性失活^［20，21］^，大幅提升传感体系的可重复使用性与长期稳定性。其次，规整的介孔结构有利于底物与产物的高效传质，可在一定程度上缓解扩散限制问题，有助于维持酶的催化效率，进而增强信号输出与检测灵敏度。此外，可调控的孔道尺寸可发挥分子筛作用，阻挡干扰物质接近酶活性中心，降低非特异性响应，提升检测的选择性^［22，23］^。上述特性使多孔框架材料在构建适用于复杂基质分析的高稳定性酶传感器方面展现出良好前景^［19］^。

本文旨在系统梳理基于多孔框架材料固定化酶的生物传感器在农药残留分析领域的研究进展。首先概述酶固定化的主要策略，包括表面吸附、后渗透及原位包埋等方法，分析不同固定化方式对酶活性、稳定性及可重复使用性的影响；其次，根据信号输出模式对固定化酶生物传感器进行分类，重点探讨比色、荧光、电化学及化学发光型传感器的构建原理及其在农产品及环境样品农药检测中的应用实例；最后，分析当前该技术在农药检测应用中面临的瓶颈与挑战，以期为开发适用于现场快速检测的高效、稳定传感平台提供理论参考。

## 1 酶固定化方法

根据酶与框架材料的结合方式，现有固定化策略主要分为表面吸附、后渗透与原位包埋法3类，各类方法在酶负载效率、催化效率、检测选择性及稳定性方面各具特点（图1）。

**图1:**
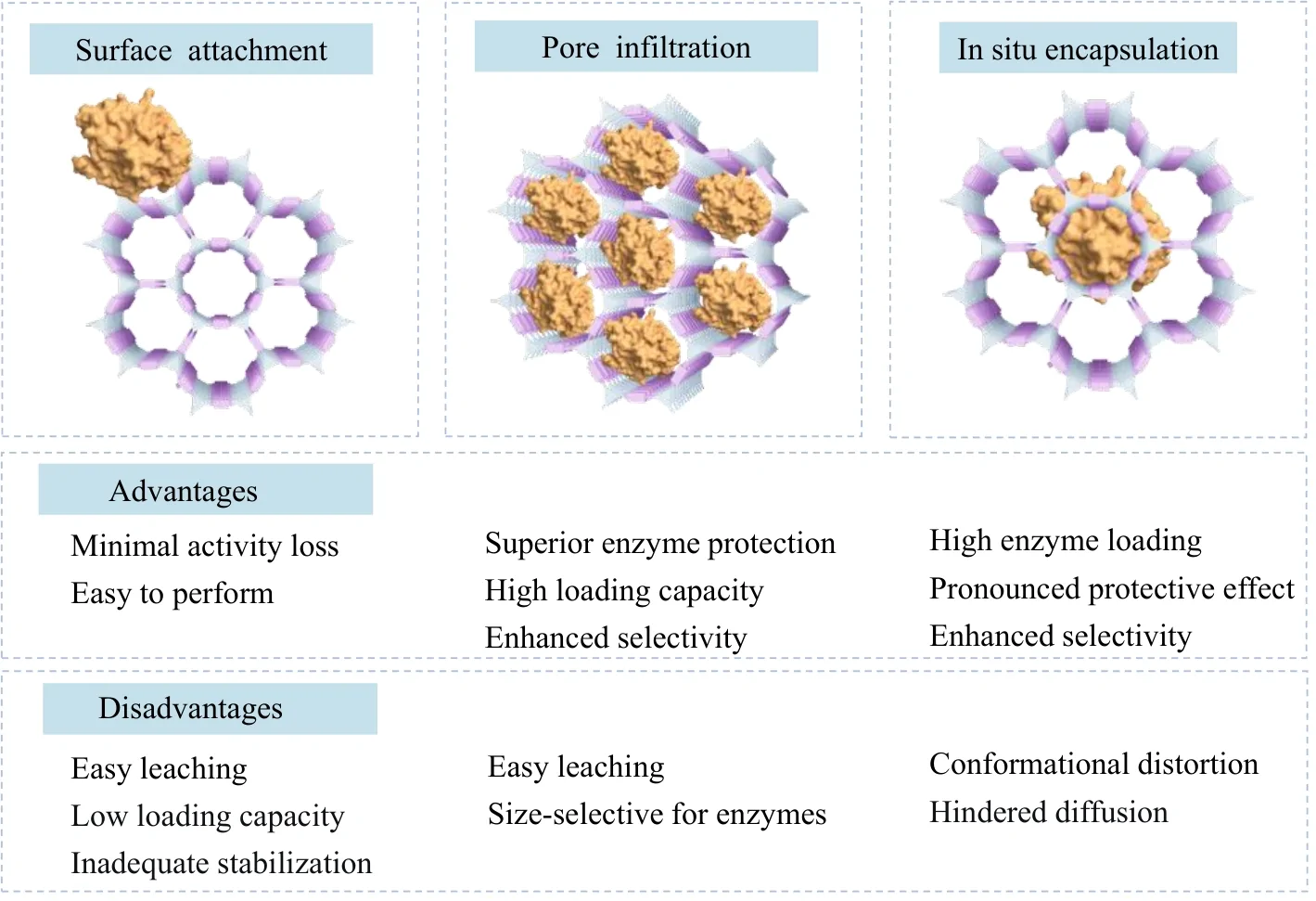
基于多孔框架材料固定化酶的策略及其各策略特点示意图

### 1.1 表面吸附

表面吸附通过非共价相互作用或共价键合将酶固定于框架材料外表面。该方法操作简便，条件温和，对酶天然构象影响较小，是目前应用最广泛的固定化策略之一。

#### 1.1.1 基于非共价相互作用的表面吸附

该策略依赖范德华力、氢键、*π-π*堆叠、疏水作用或静电吸附等弱相互作用，将酶锚定于预合成框架材料的表面。其实施条件温和，无需对酶进行化学修饰，有利于保持酶的催化活性。Ma等^［24］^通过静电配位策略将Ni^2+^修饰于ZIF-8表面，利用其与胃蛋白酶（PEP）之间的强静电相互作用实现酶的定向固定。该方法不仅操作简便，且由于弱相互作用对酶结构的扰动较小，固定化酶通常能维持较高的催化效率。从分析性能角度而言，酶的定向排布有利于活性位点充分暴露，同时表面固定缩短了底物扩散路径，减少传质阻力，有助于信号放大与检测灵敏度提升。Wu等^［25］^进一步采用氨基修饰策略构建了电化学生物传感器。与未修饰的MOF相比，NH_2_-MOF中的氨基赋予骨架更高的正电性，从而增强了载体与乙酰胆碱酯酶（AChE）之间的静电吸引作用，促进了酶的固定化过程。同时，NH_2_-MOF骨架中未配位的氧原子及（CH_3_）_2_NH_2_^+^基团亦可通过氢键与AChE形成更为稳固的结合。得益于上述多重相互作用，NH_2_-MOF传感器对草甘膦展现出宽广的线性响应范围。然而，该类体系在实际应用中仍面临稳定性挑战。pH波动、离子强度变化等环境因素易扰动非共价吸附平衡，导致酶缓慢脱落，长期使用后响应信号可出现明显衰减，进而影响传感器的长期可靠性与现场适用性。

#### 1.1.2 表面共价作用

为克服非共价吸附中酶易脱落的问题，表面共价作用策略通过形成稳定的共价键将酶固定于框架材料表面。载体上的羧基、叠氮基、环氧基等官能团可与酶表面的氨基、羧基或巯基发生特异性偶联^［26］^，实现酶的共价固定。Lu等^［27］^将葡萄糖氧化酶共价键合于环氧功能化的COFs表面，使酶负载量由物理吸附的26 mg/g提升至40 mg/g；同时，共价固定化酶经6次循环使用后活性保持接近100%，而物理吸附法仅保留65.1%的活性；60 ℃处理6 h后，共价固定化酶活性保留48.8%，物理吸附法则仅余35.6%；储存7 d后，共价固定化酶活性仍达87.4%，物理吸附法为83.9%。共价策略在重复使用性、热稳定性及储存稳定性方面均显著优于静电吸附。共价固定显著提升了传感器的操作稳定性与可重复使用性，有利于降低检测成本。然而，该方法存在局限：酶直接暴露于外部环境，易受外界条件影响而失活；固定化反应主要发生于材料外表面，内部孔道利用率低，限制了负载量的进一步提升。此外，共价修饰可能扰动酶的天然构象，导致其活性下降与选择性降低。

### 1.2 后渗透

后渗透策略主要依赖于溶液扩散作用，使酶分子进入预先合成框架材料的孔道内部。该过程在温和条件下进行，无需对酶进行化学修饰，有利于保持其天然结构与催化活性。Ma等^［28］^通过后渗透法将过氧化物酶-11（MP-11）包埋于介孔Tb-mesoMOF中，酶负载量19.1 μmol/g（为介孔二氧化硅MCM-41的5.6倍），催化底物转化效率提升至48.7%，且循环使用7次无酶浸出、活性无明显衰减，溶剂耐受性与循环稳定性远优于传统介孔二氧化硅体系。类似地，COFs凭借其可调的孔道微环境，为酶活性的调控提供了新途径。研究表明，脂肪酶PS被包埋于具有规整一维孔道的COF-OMe后，酶负载量达0.95 mg/mg，且在疏水孔道界面激活作用下催化活性大幅提升，其活性随孔道壁功能化修饰发生规律性变化，表明通过精准设计主-客体相互作用可实现酶性能的定向优化^［29］^。为克服扩散限制问题，研究者进一步开发了具有多级孔结构的框架材料，如含有相互连通的介孔或大孔的MOFs及COFs体系^［30］^。在这类材料中，大孔主要用于酶分子的负载，小孔则保障底物与产物的高效传质，从而协同提升传感性能。从分析应用的角度来看，后渗透策略不仅实现了酶的高效固载，其可精准调控的孔道尺寸还可发挥分子筛效应，选择性阻挡干扰物质接近酶活性位点，从而显著提升检测的选择性。然而，该方法对孔道尺寸的严格要求也在一定程度上限制了其适用范围，主要体现在两方面：一是仅适用于尺寸小于框架孔径的酶，二是大孔框架材料的合成通常工艺复杂，这为其规模化应用带来了挑战。

### 1.3 原位包埋

原位包埋是指在框架材料合成过程中直接将酶掺入其晶体结构的方法。该过程中，框架前驱体与酶在溶液中共同组装，材料围绕酶分子结晶生长，形成具有保护作用的刚性壳层。这一策略可有效抑制酶从载体中泄漏，显著提升酶在极端环境下的稳定性。同时，框架本身的分子筛功能得以保留，可通过阻挡干扰物质接近酶活性中心，降低非特异性背景信号，从而提高检测选择性。此外，原位包埋还可实现对尺寸大于框架本征孔径的酶的有效固定，并通常能够同时实现较高的负载量与活性保持^［31］^。

#### 1.3.1 MOFs

ZIF-8因其温和的合成条件被广泛用于酶的原位固定，目前已成功实现漆酶（Lac）^［32］^、辣根过氧化物酶（HRP）^［33］^、细胞色素C（Cyt c）^［34］^、AChE^［35］^等多种酶的封装。所得复合材料在农药及酚类检测中展现出良好的应用潜力，框架材料可有效提升酶在苛刻条件下的操作稳定性。然而，ZIF-8体系存在固有局限性：其孔窗较小（约3.4 Å），限制了大分子底物的传质；在酸性或螯合剂存在条件下，其结构稳定性较差。近期研究表明，其他MOF体系如MOF-545-Fe^［36］^、Tb-BTC^［37］^、Zn-MOF-74^［38］^、UiO-66-F_4_^［39］^等亦可实现酶的稳定封装，并在促进底物传质方面表现优异。以UiO-66-F_4_为例，研究者通过仿生矿化策略将碱性磷酸酶（ALP）与HRP共封装于该 MOFs 骨架中^［39］^。刚性的UiO-66-F_4_外壳为酶分子提供了有效的物理屏蔽，显著增强了其抵御高温与极端pH环境的能力：在60 ℃条件下，固定化ALP与HRP的相对活性分别保持在76.1%与77.7%，而游离酶仅余21.4%与39.8%；即使升温至90 ℃，两者仍可保留42.3%与50.2%的催化活性，远优于游离酶。此外，限域孔道内双酶的邻近排布有效缩短了底物扩散路径，进而大幅提升了级联反应效率。由此制备的复合材料在催化性能、选择性及操作稳定性方面均获得了显著增强。

#### 1.3.2 HOFs

氢键有机框架（HOFs）是一类由有机结构单元通过分子间氢键、*π-π*堆积等弱相互作用自组装而成的多孔晶体材料。其合成条件温和，无需金属参与，因而具备优异的生物相容性与结构可调性，为酶分子的原位固定化提供了绿色且高效的载体平台^［19］^。本课题组率先发展了一种蛋白质导向的界面组装策略，成功将多种酶原位封装于水稳性介孔HOF（HOF-101）中^［40］^。该策略不仅实现了超高的酶包埋效率，而且其介孔孔道结构有效保障了底物向酶活性中心的高效传质。进一步，借助该原位组装方法，可将两种乃至3种酶包埋于长程有序介孔骨架内。其规整的介孔通道显著加速了底物与产物在活性位点间的定向扩散，从而有力推动了级联反应的进程^［41］^。与传统的微孔ZIF-8限域体系相比，介孔HOF基级联系统大幅度提高了化学转化速率，实现多重分析物的超灵敏检测。此外，该刚性框架为多酶体系提供了物理屏蔽效应，显著提升了级联反应的稳定性与循环性能。

在此基础上，该课题组近期进一步将AChE封装于聚烯丙胺盐酸盐-HOF材料中，并将其嵌入柔性水凝胶基质，成功研制出一种能够实时传递农药残留信息的植物可穿戴传感器^［42］^。该传感器通过与智能手机集成的图像处理算法，对番茄植株表面有机磷农药的动态分布实现了原位、可视化监测，其对毒死蜱的检出限低至1 ng/mL。

#### 1.3.3 COFs

COFs凭借其共价键连接方式，通常具有优于MOFs和HOFs的化学稳定性，但其传统合成往往需要高温、有机溶剂及酸催化剂等苛刻条件，难以与酶的原位固定兼容。Chen等^［43］^提出基于MOFs牺牲模板的酶@COFs构建方法，该策略可拓展至多种COFs材料，但操作复杂性较高。该团队随后开发了以乙酸为催化剂的水相一步合成法，突破了传统策略在孔径适配性方面的限制，成功构建了一系列酶@COFs复合材料^［44］^。其结果表明，即便选用孔径匹配的NKCOF-100与MCM-41作为载体，通过物理吸附获得的酶负载量也仅分别为0.38 g/g与0.28 g/g，远低于原位组装策略下酶@NKCOF-98所实现的0.74 g/g。此外，物理吸附法制备的固定化酶泄漏率均超过65.0%，而酶@NKCOF-98在相同条件下则几乎未检测到酶泄漏现象。在后续的稳定性评估中，原位包埋策略所制备的酶@COFs生物催化剂在pH3.0、100 ℃高温及表面活性剂等严苛条件下处理后仍能维持出色的催化活性；相比之下，原位固定化的酶@MOFs体系（如ZIF-8、UiO-66）及物理吸附固定化体系在同等处理条件下均丧失了绝大部分酶活力。这主要是由于在COFs骨架形成过程中，酶分子既可嵌入颗粒表层亦可被包裹形成核壳结构，同时酶分子表面的氨基与COFs单体中的醛基发生共价缩合反应，形成化学键连接，从根本上遏制了操作过程中的酶泄漏，从而在负载量、固定化效率和稳定性方面均展现出显著优越性。本课题组建立了一种以微量离子液体（仅2 μL）为催化剂的绿色水相合成体系，在温和条件下快速实现了高结晶度酶@COFs复合材料的制备^［23］^。此外，机械化学法为酶@COFs的绿色制备提供了新思路^［45］^。该方法可最大限度减少有机溶剂与强酸碱试剂的使用，规避COFs结晶的苛刻条件，有效避免酶的变性失活。

## 2 基于固定化多孔框架材料的农药检测分析方法

基于酶固定化多孔框架材料的农药检测方法，依据信号转导机制的不同，主要可分为比色、荧光、电化学等类型，如表1所示。以下将系统阐述各类传感器的结构工作原理及性能特点，并总结其在农产品及其环境中农药快速检测中的实际应用，以期为开发高效、低成本、便携式的酶生物传感器提供理论依据与技术参考。

**表1:** 酶固定化多孔框架材料的农药检测方法

Detection method	Target	Porous framework	Enzyme	Linear range/（ng/mL）	LOD/（ng/mL）	Ref.
Colorimetric	chlorpyrifos	Zn-MOF-74	AChE	1.0‒600	1.0	［^38^］
	chlorpyrifos	PAH-HOF	AChE	1.0‒800	1.0	［^42^］
	acephate	ZIF-8	AChE/CHO/HRP	0.183‒183	0.0421	［^35^］
	DDVP	Ce/Zr-MOF	AChE/CHO	0.5‒500	0.32	［^46^］
	ethyl paraoxon	ZIF-8	AChE/HRP	6‒800	1.8	［^47^］
	MP	MPNFs	AChE/CHO/HRP	0.0224‒224	0.00717	［^48^］
	glyphosate	UiO-66-F_4_	ALP/HRP	3.38‒112.77	1.69	［^39^］
	QpE	mZIF	QpeH/AOx	3.06×10^4^‒8×10^4^	3.06×10^4^	［^49^］
	glyphosate	MOF-545-Fe	AChE	2.0‒800	0.65	［^36^］
	omethoate			0.5‒800	0.16	
	paraoxon			1.0‒800	0.32	
	imidacloprid	NSMOFs	HRP	0.02‒1.1	8.0×10^‒3^	［^50^］
	imidacloprid	MOFs-P	HRP	0.001‒500	0.0013	［^51^］
	isocarbophos	COFs-PB	HRP	0.05‒1000	0.03	［^52^］
	chlorpyrifos	HP-UiO-66-OH	Cytc	0.01‒10	4.63×10^‒3^	［^53^］
	chlorpyrifos	ZIF-8	AChE	0.35‒17.53	0.214	［^54^］
	MP	Tb-BTC	OPH	22.4‒2.24×10^5^	0.58	［^37^］
Fluorescent	MP	ZIF-8	AChE/CHO	1‒5000	0.23	［^55^］
	MP	MPNCs	AChE/CHO	0.011‒224	3.36×10^‒3^	［^56^］
	MP	UiO-66-NH_2_	OPH	10‒10^6^	10	［^57^］
Electrochemical	carbaryl	COFThi-TFPB	AChE	442.7‒1.2×10^4^	44.3	［^58^］
	carbaryl	COFDhnda-BTH	AChE	96.6‒7×10^3^	32.2	［^59^］
	glyphosate	NH_2_-IMOF	AChE	1.69×10^‒7^‒0.169	2.09×10^‒5^	［^25^］
	isocarbophos	ZIF-8	AChE	0.5‒100	0.18	［^60^］
	malathion	COF-DhaTab	AChE	0.33‒3.3×10^3^	0.165	［^61^］
Electrochemical	malathion	COFTFPB-DBD	AChE	1×10^‒6^‒0.01	3.0×10^‒7^	［^62^］
	MP	La-MOF-NH_2_	AChE	1.0×10^‒4^‒5	5.8×10^‒5^	［^63^］
	MP	p-COFs	AChE	0.426‒8.5×10^3^	0.052	［^64^］
	MP	Cu-MOF	lipase	22.4‒8.5×10^3^	15	［^65^］
	MP	ZIF-8	BCL	2.24×10^4^‒8.5×10^6^	6.27×10^4^	［^66^］
	MP	UiO-66	BCL	1.12×10^3^‒9×10^3^	188	［^67^］
	trichlorfon	COF-LZU1	AChE	0.2‒19	0.067	［^68^］
	paraoxon	Aza-COFs	AChE	10‒1000	1.4	［^69^］
	paraoxon	Cu_3_（THQ）_2_	AChE	1‒1000	0.37	［^70^］
	malathion	ZIF-8	AChE	9.9×10^‒4^‒99	3.6×10^‒4^	［^71^］
	omethoate			9.99×10^‒4^‒999	2.81×10^‒4^	
	MP	COF-Bta-NSs	AChE	5.0×10^‒4^‒10	2.04×10^‒4^	［^72^］
	paraoxon			1.0×10^‒3^‒10	7.94×10^‒4^	
	malathion			0.05‒100	5.37×10^‒3^	
	nitrofen	UiO-66	CRL	0‒2.84×10^4^	4.55	［^73^］
	nitrofen	ZIF-8	BCL	0‒3.24×10^4^	130.68	［^74^］
	nitrofen	Cu-BTC	CRL	0‒2.22×10^4^	326.71	［^75^］
Chemiluminescent	chlorpyrifos	ZIF-8	AChE	0.35‒14.02	0.046	［^54^］
	nitrofen	COF-300	CRL	5.68‒1.42×10^4^	3.13	［^76^］

DDVP： 2，2-dichlorovinyl dimethyl phosphate； MP： melamine phosphate； QpE： quizalofop-*p*-ethyl； AChE： acetylcholinesterase； CHO： cholesterol oxidase；HRP： horseradish peroxidase； ALP： alkaline phosphatase； AOx： alcohol oxidase； Cytc： cytochrome c； OPH： organophosphorus hydrolase； BCL： *Burkholderia cepacia* lipase； CRL： *Candida rugosa* lipase.

### 2.1 比色检测技术

比色检测技术因其响应迅速、操作简便、成本低廉且不依赖大型仪器，已成为农药残留现场筛查领域最成熟、应用最广的传感手段之一。该技术以酶催化反应引发的颜色变化作为核心检测信号，通过肉眼判读或便携设备读取，即可实现农药的定性及定量分析，为食品安全快速检测提供了理想方案。

在农药比色传感研究中，基于AChE抑制机理的策略最为深入。该体系利用AChE催化底物乙酰硫代胆碱（ATCh）水解生成硫代胆碱（TCh），TCh可迅速与5，5′-二硫代双（2-硝基苯甲酸）（DTNB）反应，生成在412 nm处具有强吸收的黄色2-硝基-5-硫代苯甲酸（TNB）（图2a）^［38］^。基于此，研究者将AChE原位固定于花瓣状锌基MOF（Zn-MOF-74）中，利用其多级孔道结构促进底物及产物的传质效率，使固定化酶活性维持在90%以上。毒死蜱通过抑制AChE活性，使TCh生成量减少，进而导致黄色产物浓度降低，其抑制率与毒死蜱浓度成正比，检出限为1.0 ng/mL（图2b）^［38］^。在此基础上，该课题组近期进一步将AChE封装于聚烯丙胺盐酸盐-HOF材料中，并将其嵌入柔性水凝胶基质，成功研制出一种能够实时传递农药残留信息的植物可穿戴传感器^［42］^。该传感器与智能手机图像处理算法联用，实现了对番茄植株表面有机磷农药动态分布的原位、可视化监测，对毒死蜱的检出限同样低至1 ng/mL。

**图2:**
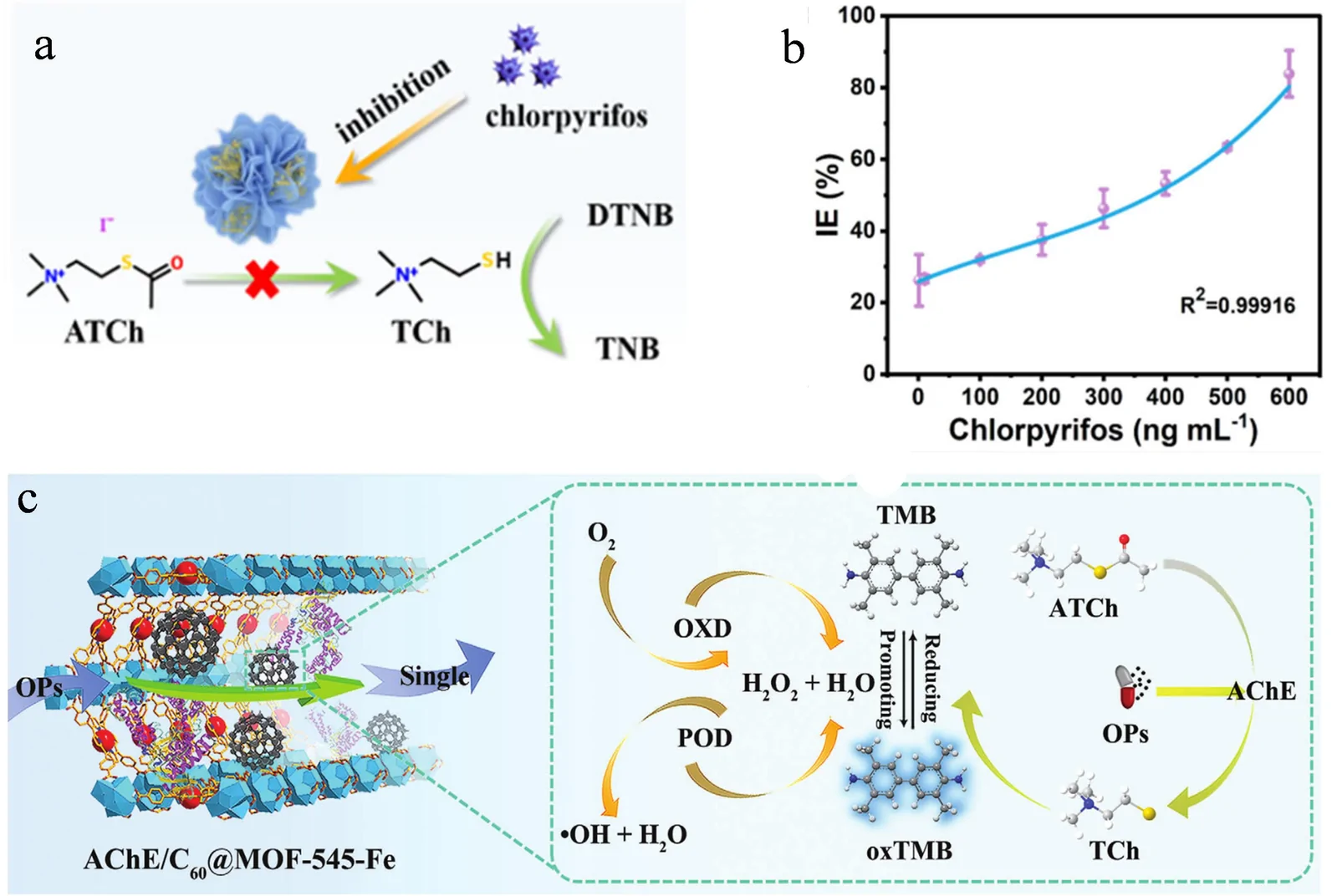
（a）基于AChE@Zn-MOF-74的比色传感示意图^［38］^；（b）毒死蜱的抑制效率标准曲线^［38］^；（c）基于AChE/C_60_@MOF-545-Fe酶-纳米酶级联流动反应器检测有机磷农药的示意图^［36］^

然而，上述基于单酶体系的比色策略在实际应用中面临显著局限，具体表现为黄色显色产物的颜色区分度有限，复杂样品基质的本底颜色易对检测信号产生光谱掩蔽与干扰。此外，单酶体系缺乏有效的信号放大机制，制约其在复杂基质中的痕量分析能力。将单酶酶促反应转化为可引发强烈颜色变化的多酶级联反应，是突破上述瓶颈的重要策略^［35，39，46-48，55，56］^。例如，Cao等^［35］^采用共沉淀技术将AChE、胆碱氧化酶（CHO）及HRP共封装于十字花状ZIF-8中，成功构建了三酶级联的比色纳米反应器。其工作机制如下：AChE催化底物乙酰胆碱（ACh）水解生成胆碱（Ch）；在ZIF-8的纳米限域空间内，Ch可被空间邻近的CHO迅速氧化生成H_2_O_2_；随后HRP利用H_2_O_2_催化TMB底物显色，产生蓝色产物。当目标农药乙酰甲胺磷存在时，其通过抑制AChE活性从而阻断级联反应，导致显色信号衰减。ZIF-8框架不仅提供稳定的限域微环境，其多孔结构将多酶分子富集于纳米限域空间内，利用邻近效应显著提升级联催化效率。该传感器对乙酰甲胺磷的检测范围为0.001~1 μmol/L，检出限为0.23 nmol/L。

传统级联比色系统高度依赖天然HRP，然而HRP成本高昂、纯化复杂且环境稳定性差。研究发现，铁基或铈基MOFs凭借配位不饱和金属中心及优异电子传递能力，可直接模拟过氧化物酶或氧化酶活性。这种将惰性载体转变为活性转导元件的策略，不仅降低了传感器构建成本，更显著提升了系统在复杂环境中的稳定性。Jin等^［46］^基于上述理念构建了生物酶-纳米酶一体化的纸基传感器。他们以Ce/Zr-MOF为类过氧化物酶核心，利用其双金属协同效应增强催化效能；通过明胶交联网络将AChE、CHO及TMB显色底物共固定于纤维素滤纸微孔中，形成高度集成的级联反应界面。该设计充分利用邻近效应加速电子传递，当有机磷农药抑制AChE活性后，级联反应受阻，体系呈现由蓝转浅的肉眼可辨颜色变化。为消除人为判读误差，研究团队开发了基于Python的图像识别算法，用户仅需智能手机拍摄即可完成光线校正、区域分割与RGB特征提取，实现对敌敌畏的精准定量，检出限为0.32 ng/mL。良好的稳定性和重现性是纸基传感器的关键评价指标。将该纸基传感器4 ℃避光存放8 w，信号仅衰减约20%，显示出优异的储存稳定性。同时，8批次独立制备的传感器对敌敌畏的检测响应高度一致，表明其批间重复性与操作可重复性良好，为多批次生产与重复使用奠定了基础。此外，铁基MOFs中Fe^2+^与Fe^3+^的电子转移特性赋予其类过氧化物酶活性。以C_60_@MOF-545-Fe为例，C_60_作为电子受体可优化卟啉配体与金属节点间的电子流转，协同增强酶活^［36］^。在此传感器中，酶促水解产物TCh可抑制TMB显色。当农药抑制AChE活性后，TCh生成减少，抑制过程减弱，将农药的毒性效应转化为正向颜色加深信号（图2c）^［36］^。进一步将YOLO v5-OPs深度学习模型集成至便携终端，实现拟合方程的直接输出，为有机磷农药的现场智能化筛查提供了解决思路。

基于AChE抑制效应的传感器虽具备广谱检测优势，能够同步响应多数有机磷及氨基甲酸酯类农药，但其非特异性识别特性限制了其在特定农药精准分析中的应用。为克服这一局限，研究者将水解酶或免疫识别元件引入多孔框架体系，借助锁钥识别机制，实现对靶标分子的特异性水解或结合，从而显著提升识别精度。针对芳氧苯氧丙酸酯类除草剂的残留检测需求，Ma等^［49］^将特异性水解酶QpeH与乙醇氧化酶共封装于具有类过氧化物酶活性的磁性ZIF-8（mZIF-8）中，构建了一种高选择性传感器。其信号转导机制如下：QpeH特异性水解芳氧苯氧丙酸酯的酯键，释放出醇类产物；随后，乙醇氧化酶将其氧化生成H_2_O_2_，进而由mZIF-8催化显色反应（图3a）^［49］^。该传感器对精喹禾灵的检测线性范围为8.2~214.6 μmol/L。此外，该传感器4 ℃储存50 d活性保持90%以上，磁性分离重复使用12次后活性仍达85%，展现出优异的长期储存稳定性与循环使用性能。

**图3:**
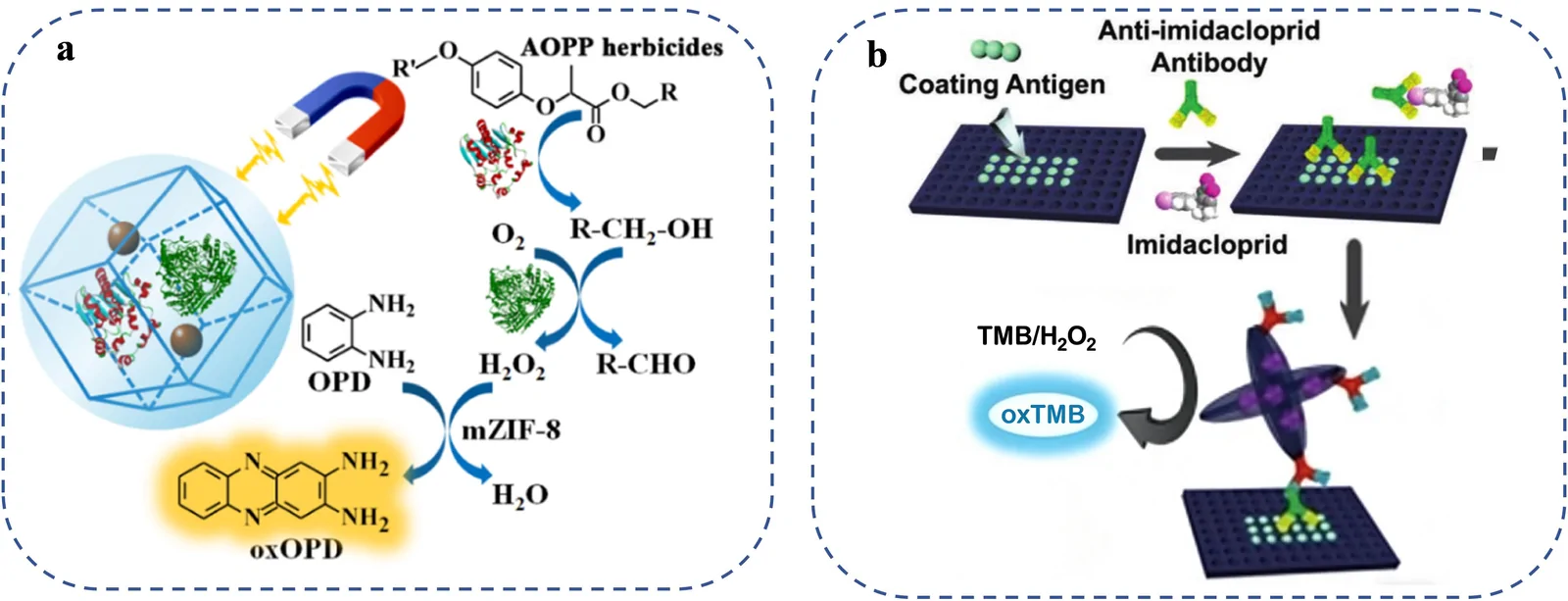
（a） QpeH/AOx@mZIF复合材料用于快速检测芳氧苯氧丙酸酯类除草剂的示意图^［49］^；（b）基于HRP@MOFs的免疫传感器用于检测吡虫啉的示意图^［50］^

水解酶能够通过特异性催化农药分子中的酯键、酰胺键等可水解键来实现高选择性识别。然而，天然酶能够直接水解的农药种类十分有限，因此其实际应用受到了较大限制。酶联免疫吸附测定策略（ELISA）将抗原-抗体反应的特异性与多孔材料性能相结合，提供了一种高适应性分析平台，仅需更换抗体即可拓展检测对象^［50-53］^。Su等^［52］^将HRP原位封装于MOFs中，构建了一种用于吡虫啉超灵敏检测的竞争型免疫传感器。如图3b所示，将包被抗原固定于96孔微孔板，使其与样品中吡虫啉竞争性结合抗吡虫啉抗体；随后，HRP@MOFs-Ab2探针通过特异性识别锚定于抗原-抗体复合物，催化TMB/H_2_O_2_显色反应^［50］^。由于探针锚定量与农药浓度呈负相关，450 nm处的吸光度随吡虫啉浓度升高而梯度下降。结合G×B颜色算法对显色图像进行解析，该平台在0.02~1.1 ng/mL范围内呈现良好线性响应，检出限低至8.0 pg/mL，展现出优异的分析性能。传统ELISA需一抗与二抗协同参与，操作烦琐且易引入非特异性吸附。为简化流程，Zhu等^［53］^构建了用于毒死蜱检测的无二抗免疫分析平台。通过对UiO-66进行合成后修饰引入高密度羟基，获得HP-UiO-66-OH材料。羟基的引入不仅优化了孔道微环境，还提升了酶活力。此外，该材料可通过Zr-O-P配位作用直接识别含磷酸酯键的毒死蜱分子。光谱分析显示，体系吸光度随毒死蜱浓度增加呈高度相关递增，在10~10 000 pg/mL范围内与浓度对数呈理想线性关系。

尽管比色传感具备操作简便、可视化与低成本优势，但其在实际现场应用中仍面临多重瓶颈。首先，显色体系易受复杂基质干扰，果蔬、食品与环境样本中的色素、多酚、糖类等物质会产生背景吸收或光谱掩蔽，导致肉眼判读与仪器定量误差显著增大。其次，可视化定量精度不足，依赖人工判读或简易设备的检测模式难以满足标准化、高通量筛查需求，检测结果的重复性有待提升。

### 2.2 荧光检测技术

荧光检测基于独特的发光机制，农药分子通过调控酶催化反应引发荧光报告分子的发射波长偏移、强度猝灭或增强。相较于比色法，荧光检测具备数量级级别的灵敏度优势、极低的本底干扰，且易于与便携式荧光读取仪或智能手机光学模块集成，满足痕量检测的实地需求。

针对高神经毒性有机磷农药甲基对硫磷的超痕量监测，Chen等^［55］^构建了基于MOF高度集成的级联纳米生物反应器。他们将AChE和CHO共封装于ZIF-8纳米孔道，利用纳米限域效应提升双酶级联催化效率；同时将谷胱甘肽修饰的金纳米簇（GSH-AuNCs）嵌入ZIF-8中，GSH-AuNCs凭借超小尺寸表现出强烈红色荧光。在该体系中，底物乙酰胆碱经AChE/CHO级联催化生成H_2_O_2_，后者在Fe^2+^介导的芬顿反应下转化为强氧化性羟基自由基，快速氧化邻近GSH-AuNCs金核，导致荧光猝灭。当甲基对硫磷抑制AChE活性后，级联反应受阻，自由基无法生成，体系荧光得以维持并随农药浓度增加呈定量恢复。这种信号增强型传感器在复杂果蔬基质中保持高信噪比，对甲基对硫磷检出限为0.23 µg/L。

除自由基介导的猝灭机制外，荧光共振能量转移（FRET）与内滤效应（IFE）的精准调控同样备受关注。将有机磷水解酶（OPH）共价固定于氨基功能化MOF表面。该固定化酶能特异催化甲基对硫磷水解，生成对硝基苯酚（PNP）^［57］^。PNP在400 nm处具有强吸收带，与香豆素1发射光谱高度重叠。MOF孔道极大缩短供受体空间距离，使香豆素1荧光随甲基对硫磷浓度增加而定量猝灭。检测线性范围为10~10^6^ ng/mL，定量限达10 ng/mL。该荧光传感器在4 ℃储存60 d后，其响应信号仍能维持初始值的近80%，表明该体系具备良好的储存稳定性与长效工作潜能。

荧光传感凭借高灵敏度与低背景干扰在痕量检测中优势突出，但规模化现场应用仍存在明显短板。首先，荧光探针易发生光漂白与自猝灭，长时间检测或强光照射下信号稳定性下降，影响检测重现性。其次，便携式荧光设备普及度低，高灵敏检测多依赖实验室大型光谱仪，现场快速检测用低成本、微型化荧光读取装置仍有待开发。

### 2.3 电化学检测技术

电化学传感器通过识别元件捕获分析物信息，将农药抑制引发的酶促反应物消耗或产物生成浓度变化，瞬间转换为电流、电位或界面阻抗等电学信号。由于信号转导发生于极薄的电极-溶液微观界面层，电化学法完全免疫于样品透光度或色泽变化的干扰，兼具低能耗与毫秒级响应特性。其次，电化学传感器易于与硅基微加工技术、微机电系统及微流控芯片高度集成，成为可穿戴农业设备及分布式传感网络的基础支撑技术。

在电化学生物传感器中，导电性MOFs或COFs可作为高效的电子传递中介体，显著提升传感性能^［25，58-75］^。例如，Niu等^［69］^通过水热法合成了富含羰基的二维导电共价有机框架（COF1）。该材料具有周期性氮杂稠合的二维*π*共轭结构，不仅赋予其优异的化学稳定性和高导电性，同时，COF1中暴露的羰基活性位点能够有效固定酶分子并维持其生物活性，还可同步富集硫胆碱，进一步增强电化学响应（图4）^［69］^。基于COF1构建的生物传感器对对氧磷检测展现出快速响应、良好的选择性与实用性，且具有优异的稳定性，检出限低至1.4 ng/mL。该电化学传感器在4 ℃中储存5周后，仍能保持初始电流响应的96.8%，展现出良好的长期稳定性。除基于AChE抑制机制检测外，基于农药直接水解的电化学检测策略亦取得重要进展。以除草醚为例，研究者将皱褶假丝酵母脂肪酶包埋于高孔隙率MOFs中，构建了具有高特异性的电化学酶传感器^［73-75］^。在该体系中，脂肪酶精准催化除草醚的醚键断裂，释放出具有电活性的酚类产物；而MOFs基底凭借其优异的导电性或氧化还原活性节点，能够高效捕获并催化产物氧化，产生显著增强的电流信号^［67，73，74］^。此外，为实现上述实验室成果向商品化分析工具的转化，Liu等^［67］^开发了一种真正可穿戴于人体的手套式生物传感器，能够实现在采摘过程中对农作物表面的农药进行原位分析。该传感系统由功能化的洋葱伯克霍尔德菌脂肪酶（BCL）@UiO-66/Pro修饰的石墨烯（LIG）三电极手套构成。这种可穿戴手套式生物传感器对甲基对硫磷的检测表现出高重复性、选择性和灵敏性，其线性检测范围为5~40 μmol/L，检出限为0.84 μmol/L。然而，电化学传感器中电极界面易污染与失稳，复杂样品中的蛋白质、多糖、腐殖酸易在电极表面吸附，导致电子传递受阻、电流信号漂移，长期使用稳定性下降。

**图4:**
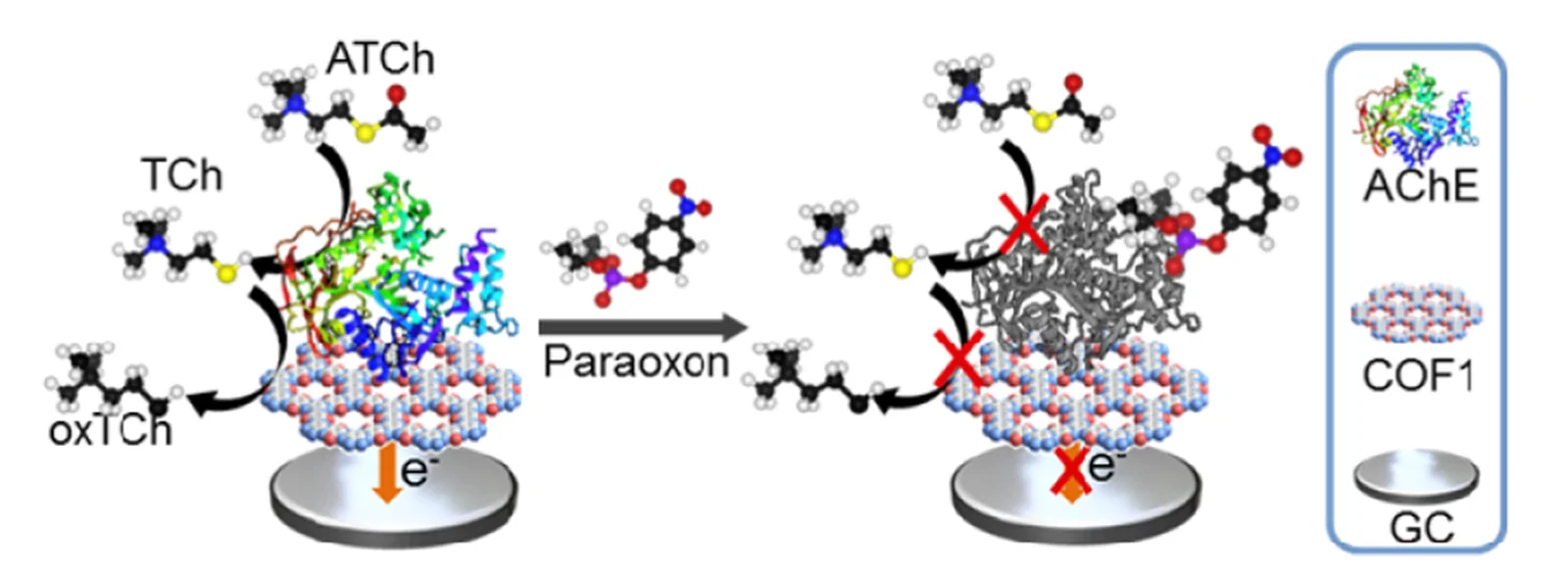
COF1/AChE检测对氧磷电化学传感原理示意图^［69］^

### 2.4 化学发光检测技术

化学发光技术通过化学反应将化学能直接转化为电子激发态，当激发态发光团辐射跃迁返回基态时释放可见光子。与传统荧光检测相比，化学发光的核心优势在于其发光过程完全由内部化学能驱动，无需外部激发光源介入，从根本上消除了背景散射光、杂散光的干扰，赋予该技术极高的信噪比。Yao等^［76］^构建了COF-300-AR@CRL复合发光平台用于痕量除草醚的检测。在该体系中，COF-300-AR框架凭借其共轭网络和富电子氨基位点，展现出优异的本征类氧化酶活性，可直接催化溶解氧氧化鲁米诺产生强烈化学发光；同时，脂肪酶（CRL）特异性催化除草醚水解断裂，释放强还原性酚类代谢物，迅速猝灭激发鲁米诺发光的自由基，导致化学发光信号显著衰减。为进一步提升复杂基质中的抗干扰能力，多通道信号交叉验证成为重要策略。Chai等^［54］^设计了具有二维纳米片形貌的V-TCPP（Fe）金属有机框架纳米酶。该材料表现出增强的类过氧化物酶、类过氧化氢酶活性及鲁米诺-H_2_O_2_化学发光性能。结果表明，其优异类酶活性源于V节点与FeTCPP配体之间的双位点电子协同效应。基于此，研究者将V-TCPP（Fe）纳米酶与AChE共封装于ZIF-8中，构建了V-TCPP（Fe）/AChE@ZIF-8限域催化体系，成功实现了有机磷农药的比色与化学发光双模态可视化检测（图5）^［54］^。结果显示，毒死蜱浓度与紫外吸光度和化学发光强度均呈现良好的线性关系。其中，比色检测在1~50 nmol/L浓度范围内线性良好，检出限为0.61 nmol/L；化学发光检测在1~40 nmol/L浓度范围内线性良好，检出限为0.13 nmol/L。双模态设计不仅提供了肉眼可见的快速半定量预警，还借助不受色泽干扰的化学发光信号实现高精度自校准定量分析，两种正交信号的交叉验证大幅提升了检测的可靠性与抗干扰能力。

**图5:**
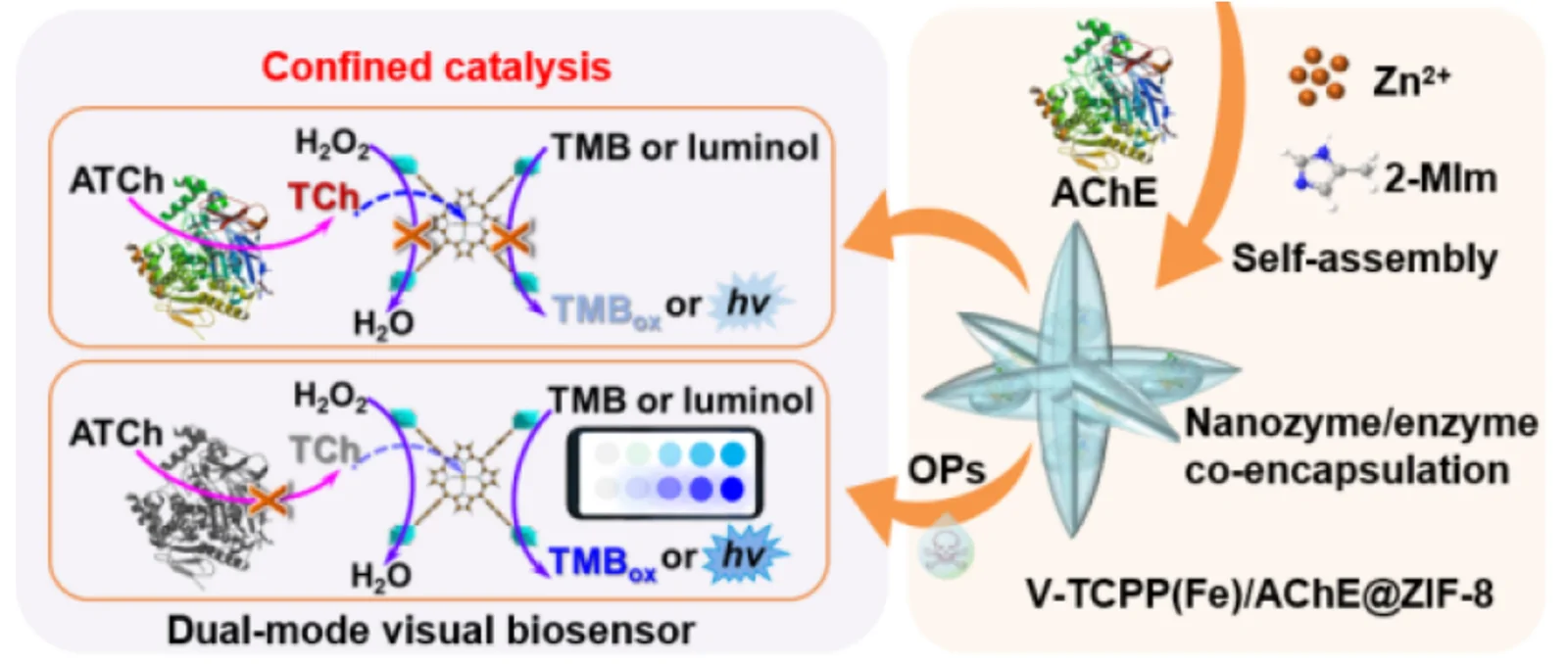
基于V-TCPP（Fe）/AChE@ZIF-8的比色与化学发光传感示意图^［54］^

化学发光因无激发光源、背景干扰极低而具备超高灵敏度，但实际应用仍受多重限制。首先，发光体系稳定性差，鲁米诺等发光试剂易受 pH、温度、溶解氧影响，信号漂移明显，检测重复性难以保证。其次，器件集成难度大，化学发光信号微弱，需要高灵敏度光电检测模块，低成本、便携式集成化设备开发滞后。

## 3 结论与展望

多孔框架材料凭借其原子级可调的拓扑孔道、超高比表面积、可精准修饰的微环境以及多模态信号转导能力，为酶基生物传感器的性能跃升提供了新平台。这类材料集酶保护、底物富集、催化增强与信号放大等功能于一体，显著提升了检测灵敏度与选择性。基于此，研究者已成功构建比色、荧光、电化学及化学发光等多模态传感体系，在有机磷和氨基甲酸酯类农药的痕量检测中展现出优异性能。然而，从实验室走向实际农药残留监测场景，仍面临一系列亟待解决的科学问题与工程挑战。

提升复杂基质中的抗干扰能力是当前亟需突破的首要问题。当前高灵敏度数据多源于理想缓冲体系，而真实农产品样本如果蔬汁、土壤浸出液中普遍存在的色素、多酚、多糖及腐殖酸等干扰物，易引发非特异性吸附、信号猝灭及背景噪声升高，导致传感器的信噪比劣化与实际检出限升高。未来研究应加强抗污损界面工程探索，通过引入两性离子聚合物或聚乙二醇刷层等亲水性屏蔽层，构筑抗非特异性吸附的稳定界面^［77］^，从源头上抑制干扰组分对传感界面的污染与信号干扰，提升传感器在复杂基质中的检测可靠性。

开发多农药同时识别的传感平台是满足实际检测需求的必要方向。实际农产品中的农药残留往往呈现多组分共存特征，而基于乙酰胆碱酯酶抑制的单通道传感器仅能反映总抑制效应，无法对特定农药进行识别与准确定量。未来应构建多通道传感器阵列^［47，78］^，利用不同识别元件对靶标分子的差异化响应产生交叉反应信号，并结合机器学习算法对响应模式进行解卷积处理。通过模式识别与数据融合技术，可实现复杂体系中多种农药残留的高通量精准识别，有效避免单一抑制检测带来的假阳性或假阴性问题^［79，80］^。

降低传感器产业化应用的材料成本是实现产品落地的经济基础。当前先进框架材料的合成多依赖昂贵有机溶剂、高温高压条件及低收率工艺，克级合成模式难以支撑一次性农残检测器件的大规模生产。未来亟需发展常温常压水相合成、机械化学球磨及连续流微反应等绿色放大技术，在保持材料结构性能的前提下降低合成成本与工艺复杂度，为农药快速检测产品的工业化生产提供经济可行的材料支撑。

将高灵敏度传感平台转化为操作简便、可规模化部署的便携式检测器件，是实现现场应用的关键一步。未来应推动柔性电子、微流控纸基芯片与物联网系统的深度融合^［81］^，结合边缘人工智能与深度学习算法，构建从数据采集、自动化分析到农药残留风险预测的全链条智能化平台。这类集成系统不仅可实现检测结果的实时输出与远程传输，还能通过数据分析挖掘污染规律，推动农药残留监测从实验室离线分析向现场主动预警的转变。

综上所述，基于多孔框架材料的酶生物传感器在农药检测灵敏度与功能性方面已取得显著进展。未来需围绕抗污界面构建、多残留识别、绿色合成及智能集成等方向持续攻关，推动该技术从实验室研究走向现场实际应用，为食品安全监测提供兼具高灵敏度与高可靠性的技术支撑。

## References

[1] Li X，Liu M，Sun W，et al . TrAC-Trends Anal Chem，2025，193： 118472

[2] Mostafalou S，Abdollahi M . Arch Toxicol，2017，91（2）： 549 10.1007/s00204-016-1849-x 27722929

[3] Pugliese P，Molto J，Damiani P，et al . J Chromatogr A，2004，1050（2）： 185 15508311

[4] Watanabe E，Kobara Y，Baba K，et al . Food Chem，2014，154： 7 10.1016/j.foodchem.2013.12.075 24518309

[5] Ghosh R K，Majumder S，Bhattacharyya A，et al . J Cleaner Prod，2021，319： 128696

[6] Tison L，Franc C，Burkart L，et al . Environ Int，2023，176： 107975 10.1016/j.envint.2023.107975 37216836

[7] Bilal M，Singh A K，Iqbal H M，et al . Chem Eng J，2023，474： 145020

[8] Pei R，Ye L，Jing C . Biosens Bioelectron，2023，229： 115244 10.1016/j.bios.2023.115244 36966618

[9] Liu S，Nie C，He F，et al . Trends Food Sci Technol，2024，150： 104597

[10] Jin J，Zhang Y F，Zheng D Y，et al . Journal of Molecular Catalysis（China），2024，38（2）： 171

[11] Devers J，Pattison D I，Hansen A B，et al . Talanta，2025，282： 127032 10.1016/j.talanta.2024.127032 39406094

[12] Feng S，Ngo H H，Guo W，et al . Bioresour Technol，2021，335： 125278 10.1016/j.biortech.2021.125278 34015565

[13] Fu Z，Yang L，Ji Y，et al . Coord Chem Rev，2026，548： 217157

[14] Côté A P，Benin A I，Ockwig N W，et al . Science，2005，310（5751）： 1166 10.1126/science.1120411 16293756

[15] Furukawa H，Cordova K E，O’Keeffe M，et al . Science，2013，341（6149）： 1230444 10.1126/science.1230444 23990564

[16] Wang B，Lin R-B，Zhang Z，et al . J Am Chem Soc，2020，142（34）： 14399 10.1021/jacs.0c06473 32786796

[17] Feng X Q，Xu Q，Zhang L H，et al ． Chinese Journal of Bioprocess Engineering，2022，20（5）： 490

[18] Mao M L，Meng L D，Gao R，et al . Chemical Industry and Engineering Progress，2023，42（5）： 2516

[19] Wang H，Kou X，Gao R，et al . Environ Sci Technol，2024，58（27）： 11869 10.1021/acs.est.4c01273 38940189

[20] Feng Y，Xu Y，Liu S，et al . Coord Chem Rev，2022，459： 214414

[21] Huang S，Chen G，Ouyang G . Chem Soc Rev，2022，51（15）： 6824 10.1039/d1cs01011e 35852480

[22] Huang W，Yuan H，Yang H，et al . Nat Commun，2023，14（1）： 3644 10.1038/s41467-023-39364-x PMC10282088 37339954

[23] Gao R，Kou X，Tong L，et al . Angew Chem Int Ed，2024，63（8）： e202319876 10.1002/anie.202319876 38183367

[24] Yang X G，Zhang J R，Tian X K，et al . Angew Chem，2023，135（7）： e202216699 10.1002/anie.202216699 36536412

[25] Wu Q，Wang Y，Wang L，et al . ACS Appl Mater Interfaces，2024，16（41）： 55802 10.1021/acsami.4c13087 39363450

[26] Oliveira F L，de Souza S P，Bassut J，et al . Chem Eur J，2019，25（69）： 15863 10.1002/chem.201903807 31596001

[27] Lu Y，Wu X，Lou H，et al . Langmuir，2025，41（18）： 11765 10.1021/acs.langmuir.5c01272 40302542

[28] Lykourinou V，Chen Y，Wang X-S，et al . J Am Chem Soc，2011，133（27）： 10382 10.1021/ja2038003 21682253

[29] Sun Q，Fu C-W，Aguila B，et al . J Am Chem Soc，2018，140（3）： 984 10.1021/jacs.7b10642 29275637

[30] Li P，Chen Q，Wang T C，et al . Chem，2018，4（5）： 1022

[31] Liang S，Wu X-L，Xiong J，et al . Coord Chem Rev，2020，406： 213149

[32] Li X，Li D，Zhang Y，et al . Nano Energy，2020，68： 104308

[33] Xu R，Liu M，Yao C，et al . ACS Appl Mater Interfaces，2025，17（3）： 5401 10.1021/acsami.4c21031 39794897

[34] Liang J，Huang Z，Wang K，et al . Talanta，2023，259： 124503 10.1016/j.talanta.2023.124503 37027932

[35] Cao X，Guo Y，Zhao M，et al . Food Chem，2022，381： 132282 10.1016/j.foodchem.2022.132282 35176684

[36] Bai Y，Nie S，Gao W，et al . Adv Funct Mater，2025，35（17）： 2419499

[37] Mehta J，Dhaka S，Bhardwaj N，et al . Sensors Actuators B： Chem，2019，290： 267

[38] Li H，Lu Q，Shi J，et al . Adv Funct Mater，2024，34（6）： 2309383

[39] Liu Y，Wang J，Qin X，et al . Food Chem，2025（493）： 145602 10.1016/j.foodchem.2025.145602 40743728

[40] Chen G，Huang S，Shen Y，et al . Chem，2021，7（10）： 2722

[41] Tang Z，Li X，Tong L，et al . Angew Chem Int Ed，2021，60（44）： 23608 10.1002/anie.202110351 34459532

[42] Li H，Liu X，Su C，et al . Adv Sci，2026，13（3）： e16233 10.1002/advs.202516233 PMC12806410 41133921

[43] Li M，Qiao S，Zheng Y，et al . J Am Chem Soc，2020，142（14）： 6675 10.1021/jacs.0c00285 32197569

[44] Zheng Y，Zhang S，Guo J，et al . Angew Chem Int Ed，2022，61（39）： e202208744 10.1002/anie.202208744 35916839

[45] Gao R，Zhong N，Tong L，et al . Cell Rep Phys Sci，2022，3（12）： 101153

[46] Jin C，Yang S，Zheng J，et al . Food Chem，2024，459： 140369 10.1016/j.foodchem.2024.140369 39002338

[47] Huang J，Jiao L，Xu W，et al . J Hazard Mater，2022，432： 128707 10.1016/j.jhazmat.2022.128707 35334265

[48] Gong C，Wang D，Zhao H . Small，2023，19（49）： 2304077 10.1002/smll.202304077 37612822

[49] Ma H，Li M，Yu T，et al . ACS Appl Mater Interfaces，2021，13（37）： 44329 10.1021/acsami.1c11815 34494423

[50] Su D，Wang T，Li A，et al . Adv Funct Mater，2022，32（36）： 2204130

[51] Yan X，Su D，Li H，et al . Adv Funct Mater，2023，33（21）： 2215192

[52] Yan X，Wei C，Su C，et al . Adv Funct Mater，2025，35（24）： 2420159

[53] Chen G，Xu W，Gu W，et al . Anal Chem，2025，97（29）： 16019 10.1021/acs.analchem.5c03157 40689565

[54] Chai H，Li Y，Yu K，et al . Anal Chem，2023，95（44）： 16383 10.1021/acs.analchem.3c03872 37881841

[55] Chen D，Wang L，Wei J，et al . Food Chem，2024，442： 138389 10.1016/j.foodchem.2024.138389 38219569

[56] Gong C，Chen B，Xing Y，et al . J Hazard Mater，2024，463： 132849 10.1016/j.jhazmat.2023.132849 37898085

[57] Mehta J，Dhaka S，Paul A K，et al . Environ Res，2019，174： 46 10.1016/j.envres.2019.04.018 31029941

[58] Luo Y，Wu N，Wang L，et al . Biosensors，2022，12（8）： 625 10.3390/bios12080625 PMC9405660 36005021

[59] Xiao Y，Wu N，Wang L，et al . Biosensors，2022，12（10）： 899 10.3390/bios12100899 PMC9599374 36291036

[60] Wen L，Wang N，Liu Z，et al . Chemosensors，2022，10（10）： 418

[61] Wang X，Yang S，Shan J，et al . Int J Electrochem Sci，2022，17（5）： 220543 10.20964/2022.05.13 PMC10276346 37360860

[62] Liang H，Wang L，Yang Y，et al . Biosens Bioelectron，2021，193： 113553 10.1016/j.bios.2021.113553 34385018

[63] Dong S，Peng L，Wei W，et al . ACS Appl Mater Interfaces，2018，10（17）： 14665 10.1021/acsami.8b00702 29620852

[64] Yang K，Zhao H，Li N，et al . Microchem J，2024，203： 110945

[65] Wang Z，Ma B，Shen C，et al . Talanta，2019，197： 356 10.1016/j.talanta.2019.01.052 30771947

[66] Ma B，Cheong L-Z，Weng X，et al . Electrochimica Acta，2018，283： 509

[67] Liu C，Wang X，Jia X，et al . Chem Eng J，2025，521： 166170

[68] Liu Y，Zhou M，Jin C，et al . Front Chem，2020，8： 643 10.3389/fchem.2020.00643 PMC7485226 33005599

[69] Niu K，Zhang Y，Chen J，et al . ACS Sens，2022，7（11）： 3551 10.1021/acssensors.2c02014 36265860

[70] Niu K，Sun P，Chen J，et al . Anal Chem，2022，94（49）： 17177 10.1021/acs.analchem.2c03766 36454682

[71] Zhao Y，Lu X and Gao F . Anal Biochem，2022，646： 114628 10.1016/j.ab.2022.114628 35257680

[72] Wei W，Zhou S，Ma D D，et al . Adv Funct Mater，2023，33（36）： 2302917

[73] Cheng Y，Lai O-M，Tan C-P，et al . ACS Appl Mater Interfaces，2021，13（3）： 4146 10.1021/acsami.0c17134 33440928

[74] Cheng Y，Ma B，Tan C-P，et al . Sensors Actuators B： Chem，2020，321： 128477

[75] Chen Y，Yuan G，Tan L，et al . J Inorg Organomet P，2022，32（11）： 4401

[76] Ma Y，Li Y . Microchimica Acta，2023，190（12）： 492 10.1007/s00604-023-06077-3 38032482

[77] Zhou L，Yang R，Li X，et al . J Am Chem Soc，2023，145（43）： 23727 10.1021/jacs.3c08256 37859408

[78] Li S，Chen X，Tian Q，et al . Anal Chem，2025，97（4）： 2537 10.1021/acs.analchem.4c06411 39835973

[79] Li Y，Chen F，Liu Y，et al . Chem Eng J，2025，511： 162115

[80] Tian T，Song D，Zhen L，et al . Biosens Bioelectron，2025，277： 117286 10.1016/j.bios.2025.117286 39985906

[81] Ibrahim H，Moru S，Schnable P，et al . ACS Sens，2022，7（8）： 2293 10.1021/acssensors.2c00834 35939805

